# Melatonin mitigates hormonal toxicity in cannabis-treated female Wistar rats: involvement of cannabinoid receptor

**DOI:** 10.1186/s42238-025-00375-8

**Published:** 2026-01-20

**Authors:** A. Oluwasola

**Affiliations:** 1 Department of Physiology, Faculty of Basic Medical Sciences, University of Ilesa, Ilesa, Osun State Nigeria; 2 Department of Nursing Science, Physiology Unit, Jimoh Babalola University, Ilorin, Kwara State Nigeria; 3 Department of Human Physiology, Faculty of Health Sciences, Al-Hikmah University, P.M.B. 1601, Ilorin, Nigeria

**Keywords:** Cannabis-sativa, Melatonin, Cannabinoid receptors, Reproductive hormones

## Abstract

**Background:**

Consumption of *Cannabis sativa* (CS), a well known psychoactive substance may impose threat on the hormonal activities of the body, hence, a protective measure is needed to prevent this threat. This study investigates the effects of melatonin and CS together with its receptors (cannabinoid receptors 1 and 2) on hormonal toxicity in female rats.

**Methods:**

Fifty female rats were assigned into ten groups of five animals each, such that the rats in groups 1,2,3,4, 5, 6, 7, 8, 9, and 10 received orally 1mL distilled water, 2 mg/kg of ethanolic extract of *Cannabis sativa* (EECS), 2 mg/kg of cannabinoid one receptor (CB_1_R) blocker (rimonabant hydrochloride), 2 mg/kg of cannabinoid two receptor (CB_2_R) blocker (am630), 2 mg/kg of CB_1_R blocker + 2 mg/kg of EECS, 2 mg/kg of CB_2_R blocker + 2 mg/kg of EECS,2 mg/kg of CB_1_R blocker + 2 mg/kg of CB_2_R blocker + 2 mg/kg of EECS,4 mg/kg of melatonin,2 mg/kg of CB_1_R blocker + 2 mg/kg of EECS + 4 mg/kg of melatoninand2mg/kg of CB_2_R blocker + 2 mg/kg of EECS + 4 mg/kg of melatonin, respectively for 14 days. Gonadotropin-releasing hormone (GnRH), follicle-stimulating hormone (FSH), luteinizing hormone (LH), estradiol (E), progesterone, and prolactin were quantified according to the instruction provided by assay kit manufacturers, using microplateimmunoenzymometric (EMA/ELISA) assays.

**Results:**

CS significantly (*p* < 0.05) decrease GnRH, FSH, LH, E, progesterone, and prolactin levels respectively when compared with the control. However, blockage of either cannabinoid receptors 1 or 2 significantly (*p* < 0.05) increase the levels of all these reproductive hormones when compared to the CS-treated group. Although, that of the former was more than the latter. All these effects were ameliorated by melatonin when the cannabinoid receptors (1 and 2) were stimulated and blocked.

**Conclusion:**

This study concluded that the gonadotoxic effects of CS could be mediated by endocrine disruption caused by cannabinoid receptors 1 and 2. In addition, CB_1_ primarily disrupts hypothalamic-pituitary-gonadal axis. Thereby causing more hormonal toxicity than CB_2_ which mainly influence hormonal imbalance indirectly through immune modulation. However, these effects could be ameliorated by melatonin. The study suggests that melatonin could be used as a supplement to prevent the gonadotoxic effects of CS.

## Introduction

Cannabis is an annual herbaceous flowering plant obtained from the flowering tops, leaves, and resin of the female plant of *Cannabis sativa* L. (family *Cannabidaceae*) (Florian et al. 1991). It is the most commonly abused illicit drug worldwide (Abdel-Salam 2016) with medicinal uses (Howlett et al. 2002). The active component, Δ^9^-tetrahydrocannabinol (Δ^9^-THC), has been used for treating migraine headache, glaucoma, nausea, and anorexia. However, effects on reproductive system have been reported. For instance, it has been shown to be spermatotoxic in male (Alagbonsi and Olayaki 2017) and ovotoxic in female (Wang et al. 2006; Oluwasola et al. 2020). The National Survey on Drug Use and Health found a 62% increase in marijuana use by pregnant women between 2002 and 2014, with the prevalence of past-month marijuana use highest in those age 18 to 25 (Young-Wolff et al. 2019). It has been reported that about 64–79% of female are cannabis users nationwide (Abuse, 2013) which can lead to pregnancy loss (Gobbi et al. 2019), low birth weight (Metz and Borgelt 2018), prematurity (Sherwood et al. 1999), intrauterine growth retardation, presence of congenital abnormalities, prenatal death and delayed the time of commencement of respiration (Gibson et al. 1983). Cannabinoids have also been reported to have negative effects on the activity of gonadotropin-releasing hormone (GnRH)-secreting neurons by direct and indirect mechanisms (Gammon et al. 2005). It also has direct effect on the pituitary gland through its receptors (Wenger et al. 1999). Moreover, it has been shown to have direct oestrogenic effect on the uterus (Wakley et al. 2014) leading to the binding of 3β-estradiol to oestrogen receptors (Wakley et al. 2014). It also has direct effect on the ovary (Takeda 2014) thus, inhibiting ovarian prostaglandin synthesis which is involved in follicular rupturing during ovulation (Torrens et al. 2020). cannabinoid one receptor (CB_1_R) blocker is mostly expressed in the central nervous system (CNS) where it mediates the central cannabinoids actions. Peripherally, its expression has been shown to be present in the reproductive tissues (Maia et al. 2020), pituitary gland, blood vessels, lung, gastrointestinal tissues, liver, adrenal gland, superior cervical ganglion, bladder, adipose tissue and immune cells (Vettor and Pagano 2009). In addition, cannabinoid receptor one (CB_1_) has been found to also be present in the ovary, uterine endometrium, testis, vas deferens, urinary bladder, and other peripheral endocrine and neurological tissues (Borowska et al. 2018). CB_2_ receptors, in contrast, have a fairly limited distribution, being found predominantly in immune cells, mast cells, splenic macrophage/monocyte preparations, immune cells (B and natural killer cells), tonsils, spleen, brain stem cells, neuronal microglia cells, hippocampus, striatum, midbrain and cerebellum (García et al. 2015), but it has now been localized in other tissues, such as central neurons, human placenta, myometrium and ovary (Han et al. 2022).

Melatonin (N-acetyl-5-methoxytryptamine) is obtained from serotonin and was first discovered and isolated in the pineal gland of cows (Ekmekcioglu 2014). It is expressed in the darkness because its highest level always coincides with the dark phase of light/dark cycle (Reiter et al. 2000). It is secreted in the pineal gland and other extra-pineal sources like retina, gut, skin, bone marrow, lymphocytes, and ovaries (Goswami and Haldar 2015). Its ability to scavenge free radicals like hydroxyl radical (^•^OH), singlet oxygen (^1^O_2_), hydrogen peroxide (H_2_O_2_), superoxide anion (O_2_^•−^), hypochlorous acid (HOCl), peroxynitrite anion (ONOO^−^), nitric oxide (NO^•^), and others in many conditions (He and He 2020) directly by free radical scavenging actions (Long et al. 2018). Studies have shown that melatonin has the capacity to mitigate hormonal toxicity possibly by its free radical scavenging ability (Tamura et al. 2013; Oluwasola and Olayaki 2020; Oluwasola et al. 2019; Jiang et al. 2021; Oluwasola et al. 2022; Oluwasola et al. 2023). Its role in reproduction has been contradictory, as both detrimental and beneficial effects have been reported (Reiter et al. 2009).

This study investigated the effects of melatonin and *Cannabis-sativa* (CS) on the reproductive hormonal toxicity in female rats. Within my research limit, I have not come across any study which has examined the major side effects of activating CB_1_ and CB_2_ receptors following the consumption of CS on the reproductive hormones and also the therapeutic effect of melatonin to preventing these side effects. This study aimed to bridge this gap.

## Materials and methods

### Animals

Fifty (50) female albino rats (160 ± 15 g) were used for this experiment. They were obtained from the Department of Biochemistry, University of Ilorin, Ilorin, Kwara State, Nigeria, housed at room temperature with unrestricted access to diet and water and maintained on a daily light/dark cycle. Principles of laboratory animal care (NIH publication No. 85 − 23, revised 1985) were followed. The experimental protocol was approved by the Ethical Committee of Al-Hikmah University, Ilorin, Nigeria with approval code “HUI/ERC/2023/095”.

### Extraction of *Cannabis sativa* leaves

Extraction of *Cannabis sativa* (*CS*), which was kindly donated by National Drug Law Enforcement Agency (NDLEA), Nigeria, for research purpose only, was done with Soxhlet apparatus by soaking 800 g of CS in 98% ethanol for 48 h, at room temperature (27 °C). It was filtered and the filtrate was poured into a round bottom conical flask which was fixed with a rotary evaporator. It was then evaporated and cooled. Air bath was used to get rid of any available solvent after the extraction process. The dried yield of the extract was 62% (Alagbonsi and Olayaki 2017).

### Experimental protocol

After 2 weeks of acclimatization, fifty female wistar rats were divided randomly into ten groups, control and nine treatment groups each of five animals. As follows:

- Group 1 (*n* = 5) received normal saline (1 ml/kg b.w)for 14 days
- Group 2 (*n* = 5) received EECS (2 mg/kgb.w) for 14 days
- Group 3 (*n* = 5) received CB_1_R blocker (rimonabant hydrochloride) (2 mg/kgb.w) for 14 days
- Group 4 (*n* = 5) received CB_2_R blocker (am630) (2 mg/kgb.w) for 14 days
- Group 5 (*n* = 5) received CB_1_R blocker (2 mg/kgb.w) and EECS (2 mg/kgb.w) for 14 days
- Group 6 (*n* = 5) received C CB_2_R blocker BRB_2_ (2 mg/kg) and EECS (2 mg/kgb.w) for 14 days
- Group 7 (*n* = 5) received CB_1_R blocker (2 mg/kgb.w) + CB_2_R blocker (2 mg/kgb.w) + EECS (2.00 mg/kgb.w) for 14 days
- Group 8 (*n* = 5) received melatonin (4 mg/kgb.w) for 14 days
- Group 9 (*n* = 5) received CB_1_R blocker (2 mg/kgb.w) + EECS (2 mg/kgb.w) + melatonin (4 mg/kgb.w) for 14 days
- Group 10 (*n* = 5) received CB_2_R blocker (2 mg/kgb.w) + EECS (2 mg/kgb.w) + melatonin (2 mg/kgb.w) for 14 days (Oluwasola et al. 2020).

NOTE:

Administration was done orally using oral cannula and once daily in the morning between 7 am and 9 am. They were checked twice daily (morning and evening) to ensure they did not lack food and water. Their cages were cleaned every three days to ensure adequate hygiene.

### Drug and assay kits

Melatonin was a product of Sigma Aldrich Company, Mannheim, Germany. The gonadotropin releasing hormone (GnRH), luteinizing hormone (LH), follicle stimulating hormone (FSH), estradiol (E), progesterone and prolactin assay kits were products of Monobind Inc., Lake Forest, California, USA. All other chemicals used were productsof Sigma Aldrich Company, Mannheim, Germany (Staff 2014).

### Preparation of serum

The female rats were sacrificed under ketamine anesthesia after the last treatment (day 15) and blood was collected from the heart puncture into sample bottles. The blood was left for 30 min to clot and thereafter centrifuged at 625×*g* for 10 min using a Uniscope Laboratory Centrifuge (Model SM800B, Surgifield Medicals, Essex, England). The serum was collected into plain bottles with the aid of a Pasteur pipette. Sera were stored in a freezer maintained at −5 ℃ and used within 12 h of preparation (Smalberger et al. 2022).

### Quantification of reproductive hormones

The serum hormone concentrations of GnRH, FSH, LH, E, progesterone and prolactin were quantified according to the instruction provided by assay kit manufacturers, using microplateimmunoenzymometric (EMA/ELISA) assays. The serum hormone concentrations were then interpolated from their respective calibration curves. ELISA analyzer (Thermo Fisher Scientific Multiskan FC) was calibrated and validated for use with rat sera. All the test kits used were products of Sigma Aldrich, South Africa (Staff 2014).

## Statistical analysis

Results were expressed as the mean ± standard error of mean. Data were analyzed using a one-way analysis of variance, followed by the LSD post-hoc test to determine significant differences in all the parameters with Students Package for Social Science, version 20.0 (SPSS Inc., Chicago, USA). Normality and homogeneity among groups were tested using Shapiro-Wilk and Bartlett’s Tests. Differences with values of *P* < 0.05 were considered statistically significant.

## Results

**Fig. 1:**
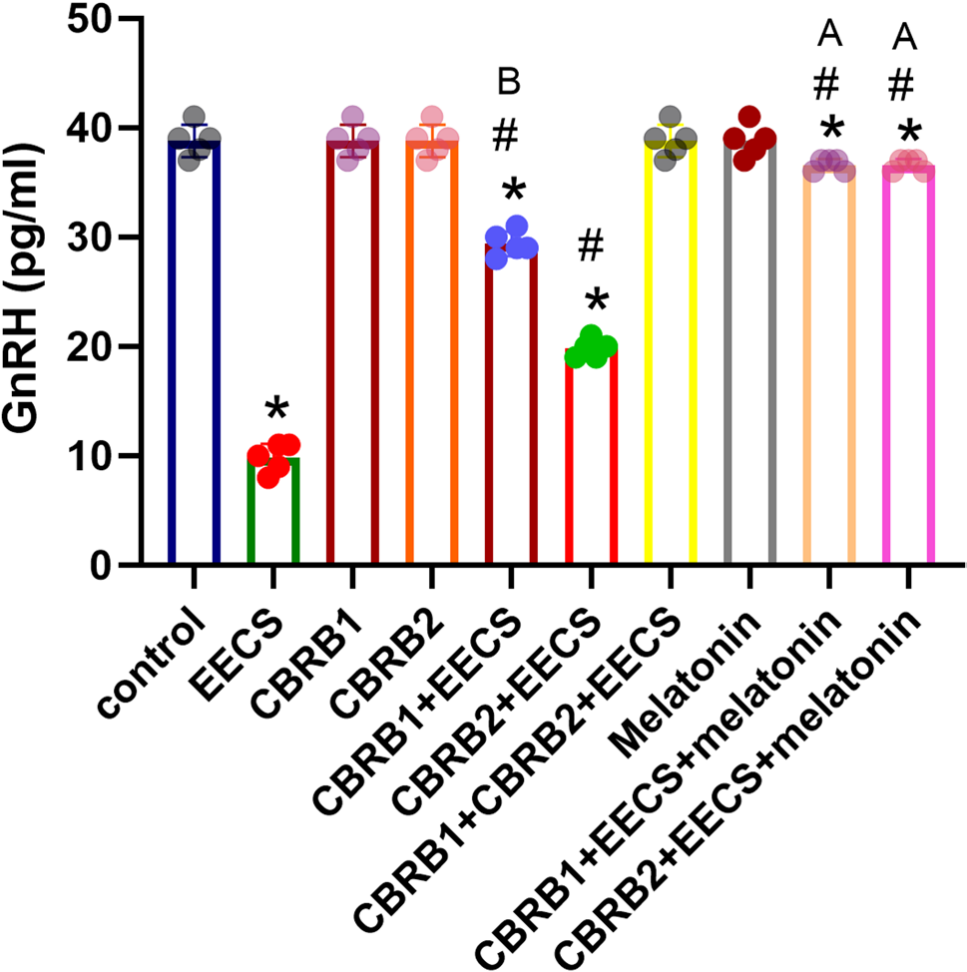
GnRH of rats that received EECS, CB_1_R blocker (rimonabant hydrochloride), CB_2_R blocker (AM630), CB_1_R blocker + Cannabis, CB_2_R blocker + Cannabis, CB_1_R blocker + CB_2_R blocker + Cannabis, melatonin, CB_1_R blocker + Cannabis + Melatonin and CB_2_R blocker + Cannabis + melatonin respectively for two weeks. Values are expressed as mean ± S.E.M; *p < 0.05 vs. Control; ^#^*p* < 0.05 vs. cannabis; ^B^*p*<0.05 vs. CB_2_R blocker + EECS; ^A^*p*<0.05 vs. CB_1_R blocker + EECS and CB_2_R blocker + EECS. The volume of rat sera analyzed was 200µL

EECS significantly (*p* < 0.05) decreased GnRH level. However, there was significant (*p* < 0.05) increased in GnRH level when the CBRs were blocked separately compared with EECS treated group. It was evident that administration of EECS with either of the blockers and melatonin significantly (*p* < 0.05) increased the GnRH level (Fig. 1).

NB: CB1R blocker–Cannabinoid receptor blocker 1; CB2R blocker – Cannabinoid receptor blocker2,

GnRH-Gonadotropin releasing hormone, EECS- Ethanolic extract of *Cannabis sativa*.

**Fig. 2:**
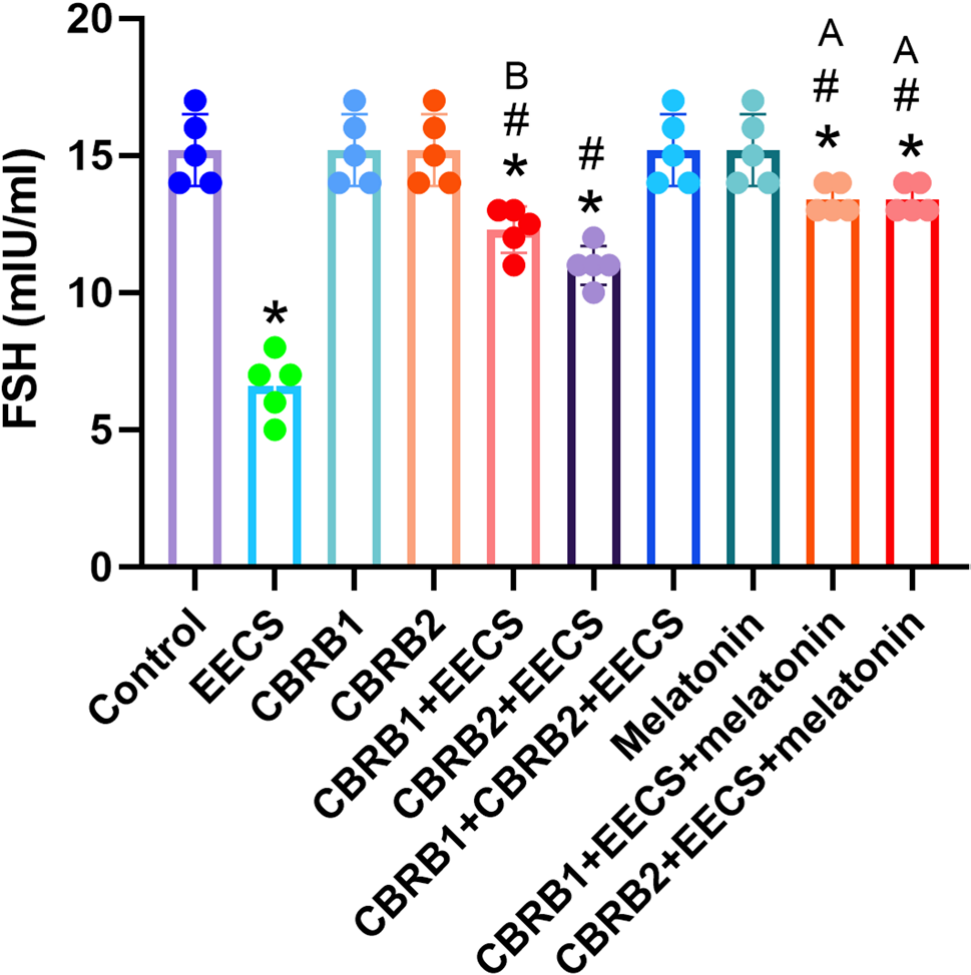
FSH of rats that received EECS, CB_1_R blocker (rimonabant hydrochloride), CB_2_R blocker (AM630), CB_1_R blocker + Cannabis, CB_2_R blocker + Cannabis, CB_1_R blocker + CB_2_R blocker + Cannabis, melatonin, CB_1_R blocker + Cannabis + Melatonin and CB_2_R blocker + Cannabis + melatonin respectively for two weeks. Values are expressed as mean ± S.E.M; *p < 0.05 vs. Control; ^#^*p* < 0.05 vs. cannabis; ^B^*p*<0.05 vs. CB_2_R blocker + EECS; ^A^*p*<0.05 vs. CB_1_R blocker + EECS and CB_2_R blocker + EECS. The volume of rat sera analyzed was 150µL

EECS significantly (*p* < 0.05) decreased FSH level. However, there was significant (*p* < 0.05) increased in FSH level when the CBRs were blocked separately compared with EECS treated group. It was evident that administration of EECS with either of the blockers and melatonin significantly (*p* < 0.05) increased the FSH level (Fig. 2).

NB: FSH-Follicle stimulating hormone.

**Fig. 3:**
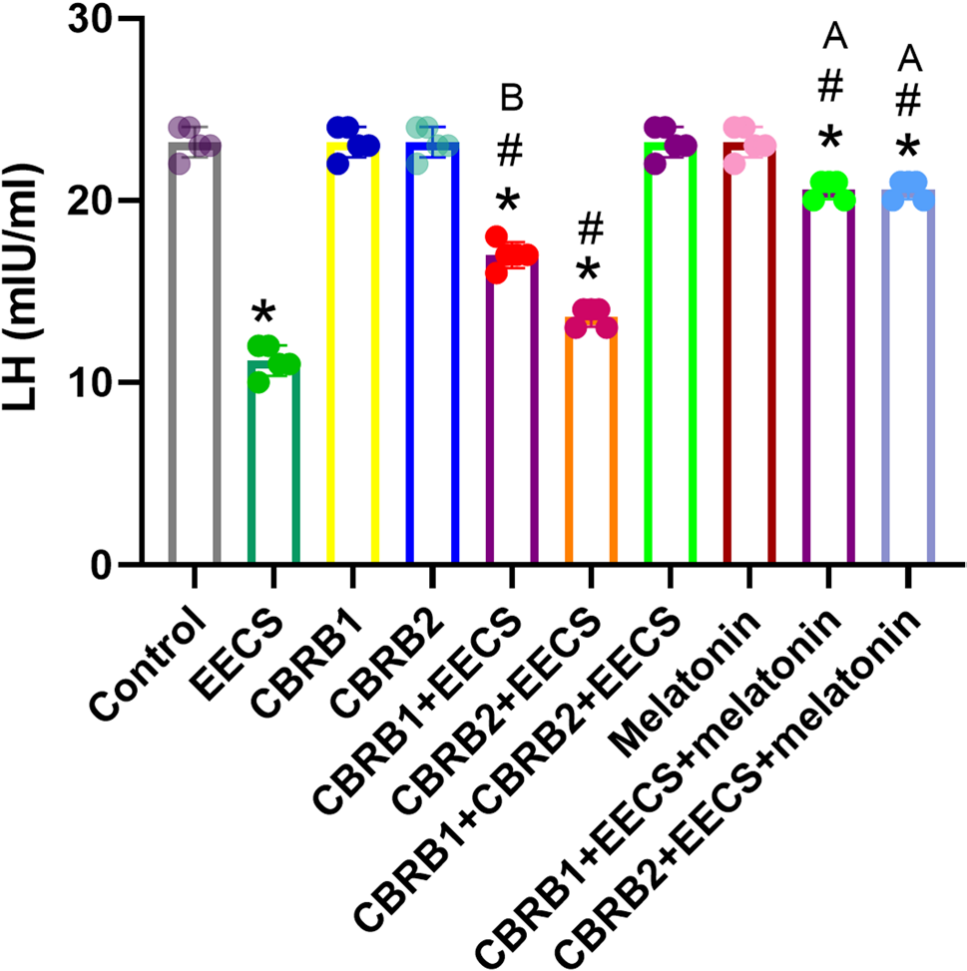
LH of rats that received EECS, CB_1_R blocker (rimonabant hydrochloride), CB_2_R blocker (AM630), CB_1_R blocker + Cannabis, CB_2_R blocker + Cannabis, CB_1_R blocker + CB_2_R blocker + Cannabis, melatonin, CB_1_R blocker + Cannabis + Melatonin and CB_2_R blocker + Cannabis + melatonin respectively for two weeks. Values are expressed as mean ± S.E.M; *p < 0.05 vs. Control; ^#^*p* < 0.05 vs. cannabis; ^B^*p*<0.05 vs. CB_2_R blocker + EECS; ^A^*p*<0.05 vs. CB_1_R blocker + EECS and CB_2_R blocker + EECS. The volume of rat sera analyzed was 200µL

EECS significantly (*p* < 0.05) decreased LH level. However, there was significant (*p* < 0.05) increased in LH level when the CBRs were blocked separately compared with EECS treated group. It was evident that administration of EECS with either of the blockers and melatonin significantly (*p* < 0.05) increased the LH level (Fig. 3).

NB: LH-Luteinizing hormone.

**Fig. 4:**
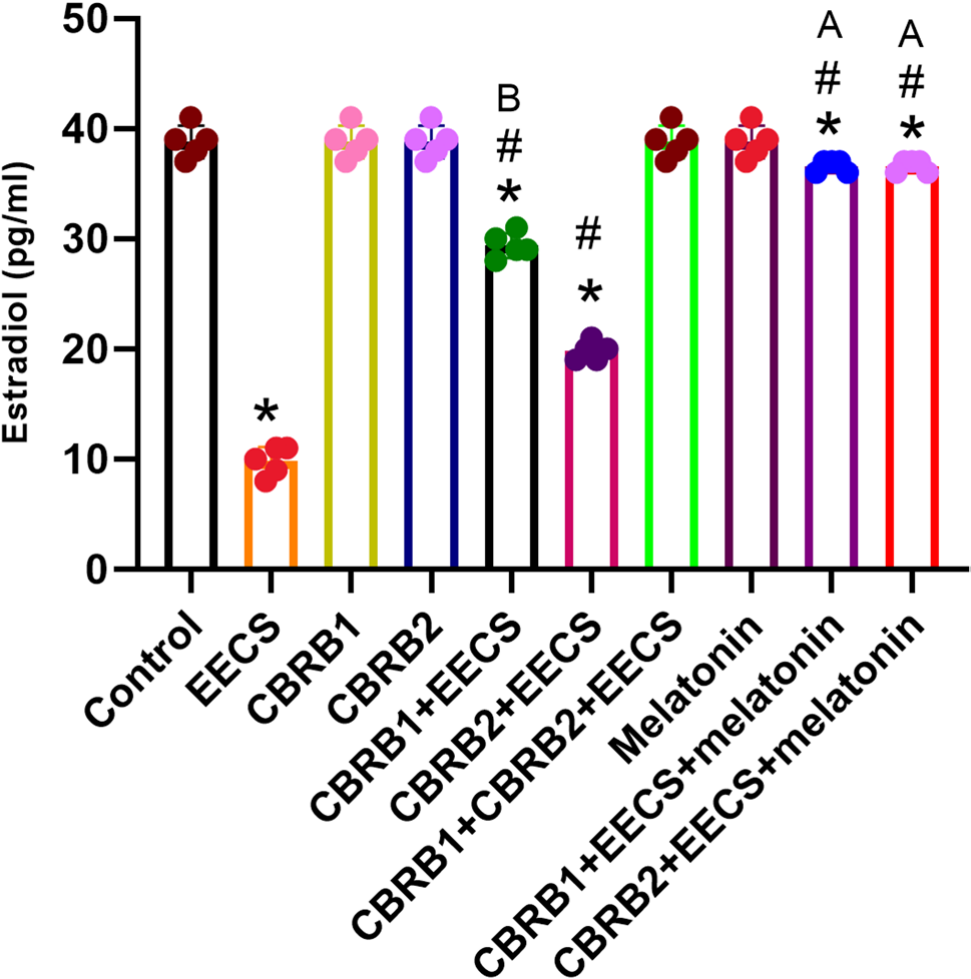
Estradiol of rats that received EECS, CB_1_R blocker (rimonabant hydrochloride), CB_2_R blocker (AM630), CB_1_R blocker + Cannabis, CB_2_R blocker + Cannabis, CB_1_R blocker + CB_2_R blocker + Cannabis, melatonin, CB_1_R blocker + Cannabis + Melatonin and CB_2_R blocker + Cannabis + melatonin respectively for two weeks. Values are expressed as mean ± S.E.M; *p < 0.05 vs. Control; ^#^*p* < 0.05 vs. cannabis; ^B^*p*<0.05 vs. CB_2_R blocker + EECS; ^A^*p*<0.05 vs. CB_1_R blocker + EECS and CB_2_R blocker + EECS. The volume of rat sera analyzed was 100µL

EECS significantly (*p* < 0.05) decreased estradiol level. However, there was significant (*p* < 0.05) increased in oestradiol level when the CBRs were blocked separately compared with EECS treated group. It was evident that administration of EECS with either of the blockers and melatonin significantly (*p* < 0.05) increased the estradiol level (Fig. 4).

**Fig. 5:**
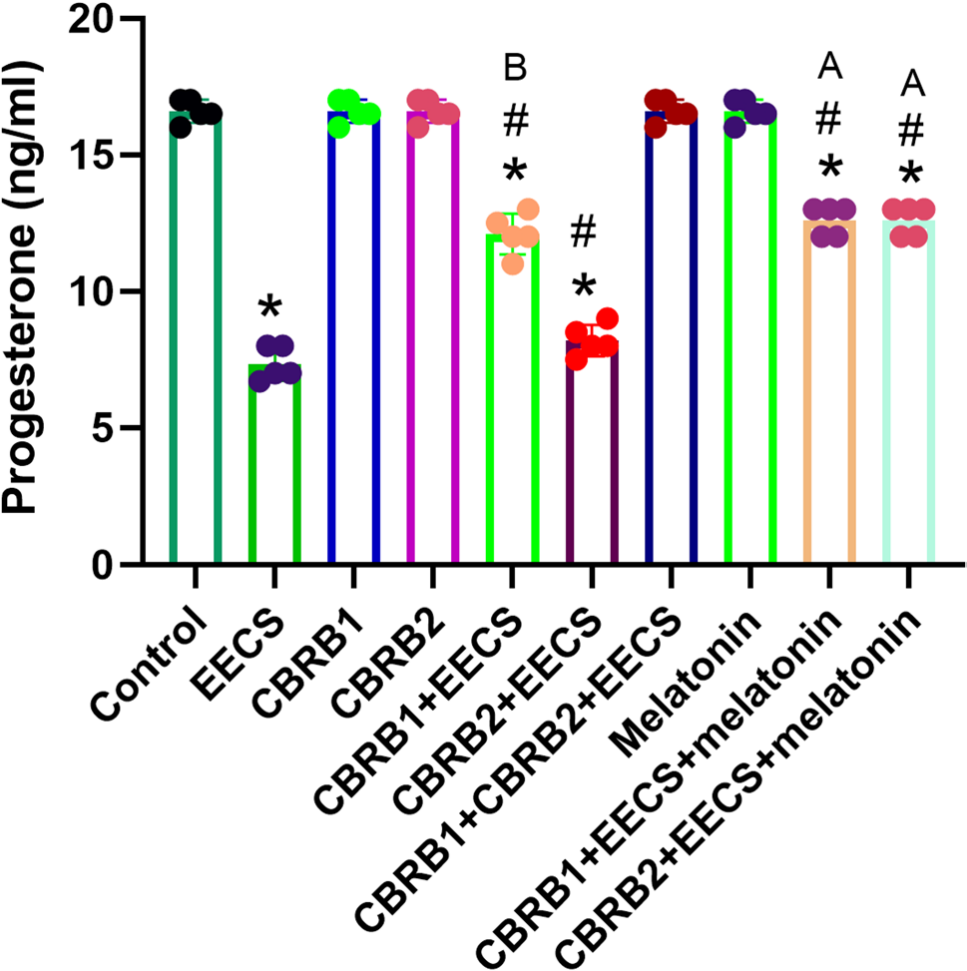
Progesterone of rats that received EECS, CB_1_R blocker (rimonabant hydrochloride), CB_2_R blocker (AM630), CB_1_R blocker + Cannabis, CB_2_R blocker + Cannabis, CB_1_R blocker + CB_2_R blocker + Cannabis, melatonin, CB_1_R blocker + Cannabis + Melatonin and CB_2_R blocker + Cannabis + melatonin respectively for two weeks. Values are expressed as mean ± S.E.M; *p < 0.05 vs. Control; ^#^*p* < 0.05 vs. cannabis; ^B^*p*<0.05 vs. CB_2_R blocker + EECS; ^A^*p*<0.05 vs. CB_1_R blocker + EECS and CB_2_R blocker + EECS. The volume of rat sera analyzed was 100µL

EECS significantly (*p* < 0.05) decreased progesterone level. However, there was significant (*p* < 0.05) increased in progesterone level when the CBRs were blocked separately compared with EECS treated group. It was evident that administration of EECS with either of the blockers and melatonin significantly (*p* < 0.05) increased the progesterone level (Fig. 5).

**Fig. 6:**
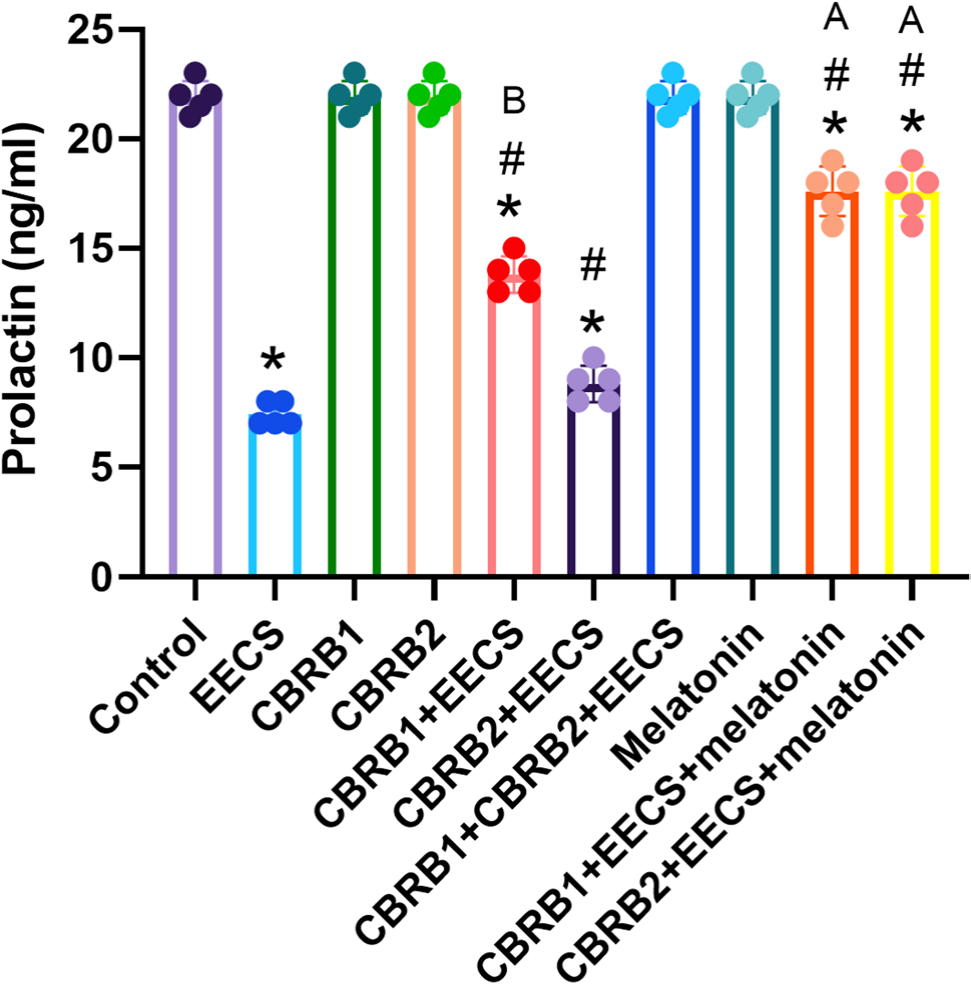
Prolactin of rats that received EECS, CB_1_R blocker (rimonabant hydrochloride), CB_2_R blocker (AM630), CB_1_R blocker + Cannabis, CB_2_R blocker + Cannabis, CB_1_R blocker + CB_2_R blocker + Cannabis, melatonin, CB_1_R blocker + Cannabis + Melatonin and CB_2_R blocker + Cannabis + melatonin respectively for two weeks. Values are expressed as mean ± S.E.M; *p < 0.05 vs. Control; ^#^*p* < 0.05 vs. cannabis; ^B^*p*<0.05 vs. CB_2_R blocker + EECS; ^A^*p*<0.05 vs. CB_1_R blocker + EECS and CB_2_R blocker + EECS. The volume of rat sera analyzed was 100µL

EECS significantly (*p* < 0.05) decreased prolactin level. However, there was significant (*p* < 0.05) increased in prolactin level when the CBRs were blocked separately compared with EECS treated group. It was evident that administration of EECS with either of the blockers and melatonin significantly (*p* < 0.05) increased the prolactin level (Fig. 6).

## Discussion

Reproductive hormones such as estrogen and progesterone play an important role in pregnancy and childbirth. These hormones increase steadily over the course of pregnancy and drop suddenly following delivery, typically returning to pre-pregnancy levels within two weeks (Agrahari and Gadagkar 2003). Given their important role in the gestational process, many experts have speculated that these reproductive hormones and other biological factors, such as stress hormones, immune and inflammatory markers, and genetic and epigenetic factors, play a role in the development of postpartum depression (García-Gómez et al. 2020).

Although research implicating the role of reproductive hormones and postpartum depression is still evolving, there is some evidence for the role of stress hormones, such as cortisol, β-endorphin, and corticotropin-releasing hormone (CRH). These hormones are implicated in depression more broadly and may be activated during pregnancy. For example, evidence supports associations between unusually high elevations of corticotropin-releasing hormone (CRH) in mid-to-late pregnancy and postpartum depressive symptoms during the initial months following delivery (García-Gómez et al. 2020). Study has shown that the entire endocannabinoid system is active at the ovarian level and CB_1_R, CB_2_R and anadamide (AEA), have been identified in ovarian tissue (Walker et al. 2019). Immunostaining shows expression of CB_1_R and CB_2_R in the medulla and cortex of the ovary. In the cortex, the receptors are expressed in the granulosa cells of primordial, primary, secondary and tertiary follicles and in the theca cells of secondary and tertiary follicles. Both receptors have also been observed in the corpus luteum and corpus albicans. In vertebrates, many authors have reported inhibitory effects exerted by endocannabinoids on the reproductive physiology in both sexes (Arrebola et al. 2010). In this study, decreased levels of GnRH, FSH, LH, estradiol and progesterone in the cannabis-treated rats could be due to the inhibitory effect of *Cannabis sativa* on the GnRH neurons in the hypothalamus (Walker et al. 2019). These effects were partially abolished when CB_1_ and CB_2_ receptors were blocked separately. However, CB_1_ receptors causes more hormonal toxicity when compared to CB_2_ receptors which could probably due to the presence of more CB_1_ receptors in the hypothalamus and in the reproductive parts which could have stimulated the action of the *cannabis sativa* on the reproductive hormones in those areas (Maia et al. 2020; Taylor et al. 2007). These effects were reduced by melatonin when the cannabiniod receptors CBRs) were stimulated and blocked. Some studies have reported increase in prolactin level following the administration of *Cannabis sativa* to both male and female rats (Tasker et al. 2015). However, the decreased in the prolactin level which was observed in the group treated with CS could be due to the stimulatory activity of dopaminergic neurons in the hypothalamus by the *CS* (Fernández-Ruiz & Ramos, 2019). In addition, this effect was partially abolished when CB_1_ and CB_2_ receptors were blocked separately. However, the effect was more in CB_1_ receptors than in CB_2_ receptors which could also be due to presence of more CB_1_ receptors in the hypothalamus and in the reproductive parts (Maia et al. 2020; Taylor et al. 2007). Activation of CB_1_ by CS primarily disrupts hypothalamic-pituitary-gonadal axis. Thereby causing more hormonal toxicity than when CS activates CB_2_, which mainly influence hormonal imbalance indirectly through immune modulation (Forner-Piquer et al. 2023). All these hormonal effects were ameliorated by melatonin when the CBRs were stimulated and blocked. The findings from this research were consistent with that of (Oluwasola and Olayaki 2020; Oluwasola et al. 2019; Oluwasola et al. 2022; Oluwasola et al. 2023) which showed that melatonin has the capacity to mitigate hormonal toxicity possibly by its free radical scavenging ability. Study has also shown that the binding of melatonin to CB_1_ receptor reduces its activity which in turn, prevents its hormonal toxicity whereas with CB_2_, melatonin decreases its effect on inflammatory-related hormonal toxicity (Mlost et al. 2020). This study has several limitations. First, it is not known whether variation in the doses of CS used could produce similar effect with the constant dose of melatonin used in this research. Second, the same dosage of melatonin was used throughout the treatment and it is not known whether its effect is dose-dependent in the CS-treated rats. Third, if treatment is done in the afternoon or at night, it is not known whether similar effect would be observed as it was done in the morning in this research.

## Conclusion

This study showed the gonadotoxic effects of CS which could be mediated by endocrine disruption. In addition, both cannabinoid receptors 1 and 2 contributed to CS-induced hormonal toxicity. Although, that of former was more than the latter. However, these effects could be ameliorated by melatonin. Since the consumption of CS is increasing globally because of its medical uses leading to its legalisation, consumption of melatonin as supplement maybe suggested for its users to prevent its hormonal toxicity. Further studies are needed to examine the longtime effects of melatonin on CS-induced reproductive hormonal toxicity in female Wistar rats as well as in other models.

## Data Availability

Available upon request.
